# Correction: Blair et al. Molecular Phylogenetic Relationships and Unveiling Novel Genetic Diversity among Slow and Pygmy Lorises, including Resurrection of *Xanthonycticebus intermedius*. *Genes* 2023, *14*, 643

**DOI:** 10.3390/genes15040451

**Published:** 2024-04-03

**Authors:** Mary E. Blair, Giang T. H. Cao, Elora H. López-Nandam, Daniel A. Veronese-Paniagua, Mark G. Birchette, Marina Kenyon, Badrul M. Md-Zain, Rachel A. Munds, K. Anne-Isola Nekaris, Vincent Nijman, Christian Roos, Hoàng M. Thach, Eleanor J. Sterling, Minh D. Le

**Affiliations:** 1 Center for Biodiversity and Conservation, American Museum of Natural History, New York, NY 10024, USA; elopez-nandam@calacademy.org (E.H.L.-N.);; 2 Department of Genetics, Vietnam National University, Hanoi 10000, Vietnam; 3 Institute for Biodiversity and Sustainability Science, California Academy of Sciences, San Francisco, CA 94118, USA; 4 The Division of Biology & Biomedical Sciences, Washington University in St. Louis, St. Louis, MO 63110, USA; 5 Department of Biology, Long Island University Brooklyn, Brooklyn, NY 11201, USA; 6 Dao Tien Endangered Primate Species Centre, Dong Nai 76000, Vietnam; 7 Faculty of Science and Technology, Universiti Kebangsaan Malaysia, Bangi Selangor 43600, Malaysia; 8 Department of Anthropology & Archeology, University of Calgary, Calgary, AB T2N 1N4, Canada; 9 Nocturnal Primate Research Group, Oxford Brookes University, Oxford OX3 0BP, UK; anekaris@brookes.ac.uk (K.A.-I.N.);; 10 School of Social Sciences and Centre for Functional Genomics, Oxford Brookes University, Oxford OX3 0BP, UK; 11 Gene Bank of Primates and Primate Genetics Laboratory, German Primate Center, Leibniz Institute for Primate Research, 37077 Göttingen, Germany; croos@dpz.eu; 12 Department of Anthropology, Vietnam National University, Hanoi 10000, Vietnam; 13 Department of Geography & Human Ecology, Rutgers, The State University of New Jersey, New Brunswick, NJ 08854, USA; 14 Faculty of Environmental Sciences, University of Science and Central Institute for Natural Resources and Environmental Studies, Vietnam National University, Hanoi 10000, Vietnam

## Error in Figure

In the original publication [1], there was a mistake in Figure 1 as published. The map did not include labels for the Paracel and Spratly Islands. The corrected Figure 1 appears below. The authors state that the scientific conclusions are unaffected. This correction was approved by the Academic Editor. The original publication has also been updated.

## Citation Correction

There was an error in one of the citations in the original publication [1]. One of the referenced citations had the incorrect year stated. 

A correction has been made to the References section:

19. Lydekker, R. On two lorises. Proc. Zool. Soc. Lond. 1904, 2, 345–346.

The authors state that the scientific conclusions are unaffected. This correction was approved by the Academic Editor. The original publication has also been updated.

## Figures and Tables

**Figure 1:**
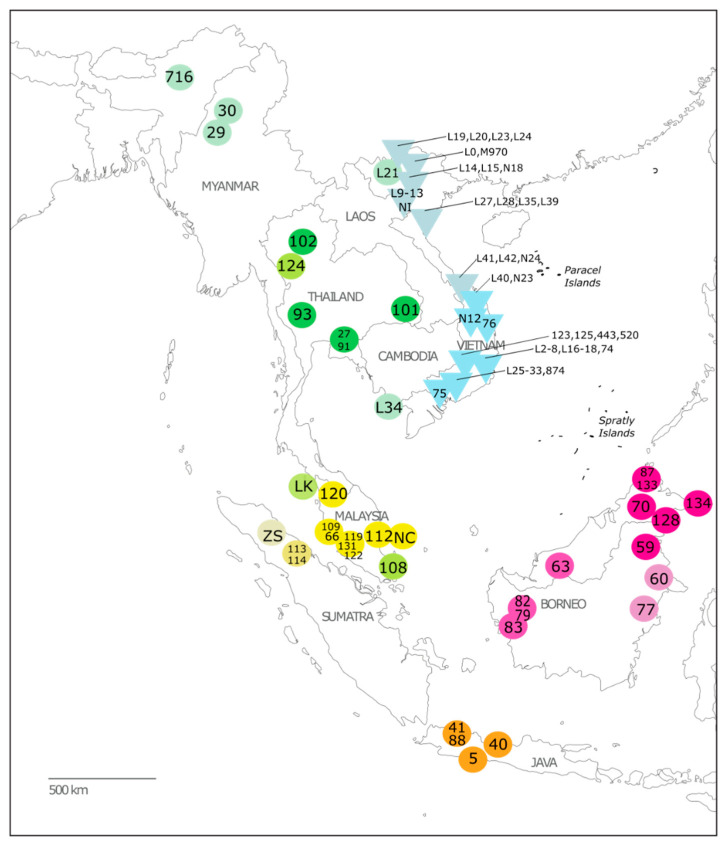
Geographic coverage of the samples included in this study for which provenance is known. Triangles represent pygmy loris samples, and circles represent slow loris samples. Colors represent clades, as shown in Figure 2. Sample IDs correspond to details provided in Table 1 and Table S1.
