# Supplementary material for: Molecular Phylogenetic Relationships and Unveiling Novel Genetic Diversity among Slow and Pygmy Lorises, including Resurrection of Xanthonycticebus intermedius
Source: Genes (Basel). 2023 Mar 3;14(3):643. doi: 10.3390/genes14030643 (PMC10048081; doi:10.3390/genes14030643)
Supplement: Supplementary file 1 [file genes-14-00643-s001.zip › Table S1.pdf]

Table S1. Sample details of additional *Xanthonycticebus* specimens used in the study for the morphological analysis and taxonomic revision, including GenBank accession numbers of additional mitochondrial sequences.

**Species name**	**Common name**	**Specimen No.**	**Map ID**	**Provider**	**Provenance**	**Sex**	**HB**	**SL**	**ML**	**PL**	**PB**	**BO**	**SB**	**BC**	**RH**	**BZ**	**Reference**	**Genbank Acc. no.(s)**
X. intermedius	Northern pygmy loris	FMNH32499	n/a	FMNH	Lai Chau, Vietnam	M	248	50.7	33.29	19.34	17.2	33.93	22.64	10.51	14.02	35.11	This study	n/a
X. intermedius	Northern pygmy loris	FMNH38911	n/a	FMNH	Thy-Ba, near Hanoi, Vietnam	F	214	50.46	30.33	19.46	16.39	32.87	21.98	9.72	14.04	32.46	This study	n/a
X. intermedius	Northern pygmy loris	FMNH38912	n/a	FMNH	Lung Linh, Ha Giang, Vietnam	M	227	50.85	31.29	21.65	18.12	33.02	21.66	10.73	13.55	34.58	This study	n/a
X. intermedius	Northern pygmy loris	HZM069	NI_pygmy	HZM	Hoà Bình, Vietnam		230	n/a	n/a	n/a	n/a	n/a	n/a	n/a	n/a	n/a	Pozzi et al. 2015	KP410626
X. intermedius	Northern pygmy loris	IZM56	n/a	IZM	Thanh tuong, Na Hang, Vietnam	F	n/a	49.81	32.54	19.89	17.47	33.83	21.75	10.49	14.01	34.7	This study	n/a
X. intermedius	Northern pygmy loris	L10	L10	Hải	Hòa Bình, Vietnam		n/a	n/a	n/a	n/a	n/a	n/a	n/a	n/a	n/a	n/a	This study	OQ518128<br>OQ555508<br>OQ555595
X. intermedius	Northern pygmy loris	L11	L11	Hải	Hòa Bình, Vietnam		n/a	n/a	n/a	n/a	n/a	n/a	n/a	n/a	n/a	n/a	This study	OQ518129<br>OQ555509<br>OQ555596
X. intermedius	Northern pygmy loris	L12	L12	Hải	Hòa Bình, Vietnam		n/a	n/a	n/a	n/a	n/a	n/a	n/a	n/a	n/a	n/a	This study	OQ518130<br>OQ555510<br>OQ555597
X. intermedius	Northern pygmy loris	L13	L13	Hải	Hòa Bình, Vietnam		n/a	n/a	n/a	n/a	n/a	n/a	n/a	n/a	n/a	n/a	This study	OQ518131<br>OQ555511<br>OQ555598
X. intermedius	Northern pygmy loris	L14	L14	Hải	Ba Vì, Vietnam		n/a	n/a	n/a	n/a	n/a	n/a	n/a	n/a	n/a	n/a	This study	OQ518132<br>OQ555512<br>OQ555572
X. intermedius	Northern pygmy loris	L15	L15	Hải	Ba Vì, Vietnam		n/a	n/a	n/a	n/a	n/a	n/a	n/a	n/a	n/a	n/a	This study	OQ518133<br>OQ555513<br>OQ555573
X. intermedius	Northern pygmy loris	L19	L19	Hải	Lào Cai, Vietnam		n/a	n/a	n/a	n/a	n/a	n/a	n/a	n/a	n/a	n/a	This study	OQ518134<br>OQ555517<br>OQ555576
X. intermedius	Northern pygmy loris	L20	L20	Hải	Lào Cai, Vietnam		n/a	n/a	n/a	n/a	n/a	n/a	n/a	n/a	n/a	n/a	This study	OQ518135<br>OQ555518<br>OQ555577
X. intermedius	Northern pygmy loris	L23	L23	M. Le	Sa Pa, Vietnam		n/a	n/a	n/a	n/a	n/a	n/a	n/a	n/a	n/a	n/a	This study	OQ518136<br>OQ555521<br>OQ555578
X. intermedius	Northern pygmy loris	L24	L24	M. Le	Sa Pa, Vietnam		n/a	n/a	n/a	n/a	n/a	n/a	n/a	n/a	n/a	n/a	This study	OQ518137<br>OQ555522<br>OQ555579
X. intermedius	Northern pygmy loris	L27	L27	M. Le	Quan Hóa - Thanh Hóa, Vietnam		n/a	n/a	n/a	n/a	n/a	n/a	n/a	n/a	n/a	n/a	This study	OQ518138<br>OQ555525<br>OQ555582
X. intermedius	Northern pygmy loris	L28	L28	M. Le	Quan Hóa - Thanh Hóa, Vietnam		n/a	n/a	n/a	n/a	n/a	n/a	n/a	n/a	n/a	n/a	This study	OQ518139<br>OQ555526

																		OQ555583
X. intermedius	Northern pygmy loris	L35	L35	M. Le	Xuân Liên - Thanh Hóa, Vietnam		n/a	n/a	n/a	n/a	n/a	n/a	n/a	n/a	n/a	n/a	This study	OQ518140 OQ555533 OQ555589
X. intermedius	Northern pygmy loris	L39	L39	M. Le	Thanh Hóa, Vietnam		n/a	n/a	n/a	n/a	n/a	n/a	n/a	n/a	n/a	n/a	This study	OQ518141 OQ555534 OQ555590
X. intermedius	Northern pygmy loris	L9	L9	Hải	Hòa Bình, Vietnam		n/a	n/a	n/a	n/a	n/a	n/a	n/a	n/a	n/a	n/a	This study	OQ518126 OQ555507 OQ555594
X. intermedius	Northern pygmy loris	HZM71	N18	HZM	Ba Vi, Ha Tay, Vietnam	F	n/a	44.08	27.04	n/a	16.51	28.68	n/a	9.28	9.32	27.95	This study	n/a
X. intermedius	Northern pygmy loris	HZM970	n/a	HZM	Lục Yên, Yên Bái, Vietnam	M	n/a	48.95	30.11	19.45	17.39	31.34	20.63	10.32	12.02	31.34	This study	n/a
X. intermedius	Northern pygmy loris	MNHN1892-1531	n/a	H. d'Orléans	Cho-Bo, Vietnam		n/a	50.97	30.38	17.96	17.14	35.77	23.51	10.92	13.79	35.72	This study	n/a
X. intermedius	Northern pygmy loris	MNHN1892-1532	n/a	H. d'Orléans	Cho-Bo, Vietnam		n/a	49.26	32.04	17.54	16.39	34.2	22.8	10.74	14.23	35.32	This study	n/a
X. pygmaeus	Southern pygmy loris	MNHN1929-454	n/a	J. Delacour	Kon Tum, Vietnam		n/a	50.93	32.61	17.61	17.43	32.36	23.77	10.34	11.87	33.97	This study	n/a
X. pygmaeus	Southern pygmy loris	443/X84	443	DLM	Lâm Đồng, Vietnam		n/a	54.73	35.31	n/a	18.4	36.42	n/a	11.47	15.71	37.34	This study	n/a
X. pygmaeus	Southern pygmy loris	492/MSx85	492	DLM	Lâm Đồng, Vietnam	M	n/a	n/a	34.72	20.5	17.56	34.02	20.33	10.73	13.56	33.1	This study	n/a
X. pygmaeus	Southern pygmy loris	520/MSx87	520	DLM	Lâm Đồng, Vietnam		n/a	54.91	34.33	20.7	18.99	37.95	25.69	11.47	14.87	37.86	This study	n/a
X. pygmaeus	Southern pygmy loris	FMNH33504	n/a	FMNH	near Saigon, Vietnam		n/a	51.52	n/a	20.39	18.52	34.39	22.08	11.96	15.29	35.71	This study	n/a
X. pygmaeus	Southern pygmy loris	FMNH46824	n/a	FMNH	Buon Ma Thuot, Dak Lak, Vietnam	M	268	51.81	33.7	20.73	18.25	32.92	24.28	10.73	14.58	36.51	This study	n/a
X. pygmaeus	Southern pygmy loris	FMNH46825	125	FMNH	Buon Ma Thuot, Dak Lak, Vietnam	F	270	54.64	33.64	20.82	18.26	35.7	23.39	10.87	16.54	37.97	This study	OQ518107
X. pygmaeus	Southern pygmy loris	FMNH46826	n/a	FMNH	Buon Ma Thuot, Dak Lak, Vietnam	M	263	53.32	35.38	22.91	17.45	35.99	22.9	10.84	14.84	38.5	This study	n/a
X. pygmaeus	Southern pygmy loris	FMNH46827	n/a	FMNH	Buon Ma Thuot, Dak Lak, Vietnam	M	265	55.56	34.76	22.77	17.72	35.79	22.34	11.04	15.97	37.5	This study	n/a
X. pygmaeus	Southern pygmy loris	FMNH46828	123	FMNH	Buon Ma Thuot, Dak Lak, Vietnam		256	n/a	n/a	n/a	n/a	n/a	n/a	n/a	n/a	n/a	This study	OQ518106
X. pygmaeus	Southern pygmy loris	FMNH46829	n/a	FMNH	Buon Ma Thuot, Dak Lak, Vietnam	F	221	55.48	34.6	21.95	19.69	34.49	23.21	11.42	15.12	38.1	This study	n/a
X. pygmaeus	Southern pygmy loris	HZM1011	N20	Vũ Ngọc Thành	Cánh Rơi, Mã Đà, Đồng Nai, Vietnam		n/a	n/a	n/a	n/a	n/a	n/a	n/a	n/a	n/a	n/a	This study	OQ555473 OQ555600
X. pygmaeus	Southern pygmy loris	IZM1879	n/a	Dinh Van C.	An Khê, Gia Lai, Vietnam		n/a	52.09	32.74	20.02	18.51	na	24.06	10.62	14.96	na	This study	n/a
X. pygmaeus	Southern pygmy loris	L1	L1	M. Blair	Lâm Đồng, Vietnam		n/a	n/a	n/a	n/a	n/a	n/a	n/a	n/a	n/a	n/a	This study	OQ518108 OQ555499 OQ555569

X. pygmaeus	Southern pygmy loris	L16	L16	Hải	Nha Trang, Vietnam		n/a	n/a	n/a	n/a	n/a	n/a	n/a	n/a	n/a	n/a	This study	OQ518116<br>OQ555514<br>OQ555599
X. pygmaeus	Southern pygmy loris	L17	L17	Hải	Nha Trang, Vietnam		n/a	n/a	n/a	n/a	n/a	n/a	n/a	n/a	n/a	n/a	This study	OQ518117<br>OQ555515<br>OQ555574
X. pygmaeus	Southern pygmy loris	L18	L18	Hải	Nha Trang, Vietnam		n/a	n/a	n/a	n/a	n/a	n/a	n/a	n/a	n/a	n/a	This study	OQ518118<br>OQ555516<br>OQ555575
X. pygmaeus	Southern pygmy loris	L25	L25	M. Kenyon	Đào Tiên, Cat Tien, Vietnam		n/a	n/a	n/a	n/a	n/a	n/a	n/a	n/a	n/a	n/a	This study	OQ518119<br>OQ555523<br>OQ555580
X. pygmaeus	Southern pygmy loris	L26	L26	M. Kenyon	Đào Tiên, Cat Tien, Vietnam		n/a	n/a	n/a	n/a	n/a	n/a	n/a	n/a	n/a	n/a	This study	OQ518120<br>OQ555524<br>OQ555581
X. pygmaeus	Southern pygmy loris	L29	L29	M. Kenyon	Đào Tiên, Cat Tien, Vietnam		n/a	n/a	n/a	n/a	n/a	n/a	n/a	n/a	n/a	n/a	This study	OQ518121<br>OQ555527<br>OQ555584
X. pygmaeus	Southern pygmy loris	L3	L3	M. Blair	Lâm Đồng, Vietnam		n/a	n/a	n/a	n/a	n/a	n/a	n/a	n/a	n/a	n/a	This study	OQ518110<br>OQ555501<br>OQ555570
X. pygmaeus	Southern pygmy loris	L30	L30	M. Kenyon	Đào Tiên, Cat Tien, Vietnam		n/a	n/a	n/a	n/a	n/a	n/a	n/a	n/a	n/a	n/a	This study	OQ518122<br>OQ555528<br>OQ555585
X. pygmaeus	Southern pygmy loris	L31	L31	M. Kenyon	Đào Tiên, Cat Tien, Vietnam		n/a	n/a	n/a	n/a	n/a	n/a	n/a	n/a	n/a	n/a	This study	OQ518123<br>OQ555529<br>OQ555586
X. pygmaeus	Southern pygmy loris	L32	L32	M. Kenyon	Đào Tiên, Cat Tien, Vietnam		n/a	n/a	n/a	n/a	n/a	n/a	n/a	n/a	n/a	n/a	This study	OQ518124<br>OQ555530<br>OQ555587
X. pygmaeus	Southern pygmy loris	L33	L33	M. Kenyon	Đào Tiên, Cat Tien, Vietnam		n/a	n/a	n/a	n/a	n/a	n/a	n/a	n/a	n/a	n/a	This study	OQ518125<br>OQ555531<br>OQ555588
X. pygmaeus	Southern pygmy loris	L4	L4	M. Blair	Lâm Đồng, Vietnam		n/a	n/a	n/a	n/a	n/a	n/a	n/a	n/a	n/a	n/a	This study	OQ518111<br>OQ555502<br>OQ555591
X. pygmaeus	Southern pygmy loris	L5	L5	M. Blair	Lâm Đồng, Vietnam		n/a	n/a	n/a	n/a	n/a	n/a	n/a	n/a	n/a	n/a	This study	OQ518112<br>OQ555503<br>OQ555592
X. pygmaeus	Southern pygmy loris	L7	L7	M. Blair	Lâm Đồng, Vietnam		n/a	n/a	n/a	n/a	n/a	n/a	n/a	n/a	n/a	n/a	This study	OQ518114<br>OQ555505<br>OQ555571
X. pygmaeus	Southern pygmy loris	L8	L8	M. Blair	Lâm Đồng, Vietnam		n/a	n/a	n/a	n/a	n/a	n/a	n/a	n/a	n/a	n/a	This study	OQ518115<br>OQ555505<br>OQ555593
X. pygmaeus	Southern pygmy loris	HZM758	n/a	HZM	Ro Coi, Sa Thay, Kon Tum, Vietnam	F	n/a	47.65	30.91	19.03	17.11	31.46	20.86	10.61	12.51	31.44	This study	n/a
X. pygmaeus	Southern pygmy loris	HZM759	N12	HZM	Ro Coi, Sa Thay, Kon Tum, Vietnam	M	260	50.75	31.88	19.44	17.05	34.96	22.56	11.21	13.54	36	This study	n/a

X. pygmaeus	Southern pygmy loris	HZM760	n/a	HZM	Ro Coi, Sa Thay, Kon Tum, Vietnam	F	280	48.05	30.49	18.59	17.5	32.88	20.39	10.24	12.1	33.04	This study	n/a
X. pygmaeus	Southern pygmy loris	HZM873	n/a	HZM	Ro Coi, Sa Thay, Kon Tum, Vietnam	F	320	49.4	31.53	n/a	19.32	34.36	n/a	11.07	13.5	34.75	This study	n/a
X. pygmaeus	Southern pygmy loris	HZM874	874	HZM	Tay Nguyen, Vietnam		n/a	50.53	31.42	19.19	17.56	33.55	n/a	10.5	13.5	34.2	This study	n/a
X. pygmaeus	Southern pygmy loris	NMNH256913	75	NMNH	Dong Nai, Bien Hoa Trang Bom, Vietnam		n/a	n/a	31.91	19.43	16.16	33.57	n/a	9.16	13.77	35.93	This study	OQ518104
X. pygmaeus	Southern pygmy loris	NMNH258234	74	NMNH	Lam Dong, Bao Loc Township, Vietnam		n/a	n/a	33.26	18.32	17.96	35.45	25.22	9.54	14.57	36.54	This study	OQ518103
X. pygmaeus	Southern pygmy loris	NMNH357750	76	NMNH	Binh Dinh an Khe, Vietnam		n/a	n/a	n/a	n/a	n/a	n/a	n/a	n/a	n/a	n/a	This study	OQ518105 OQ555495 OQ555568
X. sp++	pygmy loris	AMNH87282	64	AMNH	Plateau Bolovens, Laos	M	n/a	52.58	32.81	20.05	17.18	34.25	23.23	11.1	13.12	35.56	This study	n/a
X. sp++	pygmy loris	FMNH37986	137	FMNH	Thateng, Plateau Bolovens, Balikhamzay Prov, 90 km NE Pakse, Laos	M	255	50.09	29.85	16.85	17.5	33.44	21.75	8.66	12.94	34.94	This study	n/a

Map ID refers to ID on Figure 1 map or on Figure S5 tree (if shown).
Species and common names are following the taxonomic revision presented here.
+A skull measurement was included in the original description (55) but the skull is no longer available at HZM for remeasurement to include for comparison here.
++We excluded samples from Plateau Bolovens, Laos from any comparisons, as species genetic ID could not be confirmed for them, but they were included in tests for sexual dimorphism.

Abbreviations:

- FMNH: Field Museum of Natural History, Chicago, USA
- HZM: Hanoi Zoological Museum, Hanoi, Vietnam
- NMNH: National Museum of Natural History, Washington, D.C., USA
- DLM: Da Lat Biological Museum, Da Lat, Vietnam
- IZM: IEBR Zoological Museum, Hanoi, Vietnam
- AMNH: American Museum of Natural History, New York, NY, USA
- MNHN: Muséum National d’Histoire Naturelle, Paris, France
- HB: Total Head Body Length
- SL: Greatest Skull Length
- ML: Mandible Length
- PL: Palate Length
- PB: Palate Breadth

- BO: Biorbital Breadth
- SB: Staphylion to basion length
- BC: Bicanine Breadth
- RH: Ramus Height
- BZ: Bizygomatic breadth
