# Supplementary material for: Molecular Phylogenetic Relationships and Unveiling Novel Genetic Diversity among Slow and Pygmy Lorises, including Resurrection of Xanthonycticebus intermedius
Source: Genes (Basel). 2023 Mar 3;14(3):643. doi: 10.3390/genes14030643 (PMC10048081; doi:10.3390/genes14030643)
Supplement: Supplementary file 1 [file genes-14-00643-s001.zip › Table S2.pdf]

Table S2. Results of PCA comparing cranial measurements of *Xanthonycticebus* specimens (variable loadings, percentage variance, and standard deviation), with measurement definitions.

Variable	PC1	PC2	PC3	PC4	Measurement Definition
Greatest Skull Length	0.39128482	-0.0028046	0.01567475	-0.0841814	Rhinion to opisthocranion
Mandible length	0.35420213	-0.1021822	0.12082556	0.02885784	Gonion to infradentale
Palate length	0.34627005	-0.6091664	-0.0952445	0.14378557	Prosthion to staphylion
Palate breath (widest)	0.31572752	-0.0583725	-0.3487849	-0.8260013	between upper outer alveolar M1 junctions
Biorbital Breath	0.3030862	0.26352539	0.16258894	0.19386051	Maximum horizontal distance between outer margins of orbital rims
Staphylion to basion length	0.32991372	0.67398122	-0.1211484	0.06187643	Staphylion to basion
Bicanine breath	0.3118557	-0.114975	-0.5874765	0.49717466	Maximum breadth of muzzle across canine alveoli.
Ramus Height	0.26258232	-0.2270488	0.62200739	-0.0194368	Maximum height of the mandibular ramus
Bizygomatic breath	0.36734517	0.16307415	0.28513003	0.0125408	Greatest distance between the outer margins of the zygomatic arches
Standard Deviation	2.2202	0.965	0.84038	0.67857	
Percentage of Variance	0.6356	0.1201	0.09106	0.05937	
Cumulative Proportion of Variance	0.6356	0.7557	0.84676	0.90613	
