# Supplementary material for: Molecular Phylogenetic Relationships and Unveiling Novel Genetic Diversity among Slow and Pygmy Lorises, including Resurrection of Xanthonycticebus intermedius
Source: Genes (Basel). 2023 Mar 3;14(3):643. doi: 10.3390/genes14030643 (PMC10048081; doi:10.3390/genes14030643)
Supplement: Supplementary file 1 [file genes-14-00643-s001.zip › Table S3.pdf]

Table S3a. Primers Used In this Study

Locus	Primer	Sequence	Reference
**CO1**	5288F	CACCTCGAGGCCTGGTAAAAAGGG	This Study
	5509R	GGCTGACCTAGCTCTGCTCG	This Study
	5477F	AAGTTTGCTAATCCGAGCAGAG	This Study
	5704R	GCCGGCTCCGGCCTCAACTA	This Study
	5605F	GGGGCTTCGGGAACTGATTA	This Study
	5841R	CCCCTGCTAAGTGAAGGGAGA	This Study
	5821F	TCTCCCTTCACTTAGCAGGGG	This Study
	6052R	CCTCCTGCCGGATCAAAGAAT	This Study
	6002F	GCTGCTAACAGACCGTAACCT	This Study
	6242R	CCACACAATGAAGCCCAGGA	This Study
**Cytb**	L14724	CGAAGCTTGATATGAAAAACCATCGTTG	1
	H15149	AAACTGCAGCCCCTCAGAATGATATTTGTCCTCA	1
	L15162	GCAAGCTTCTACCATGAGGACAAATATC	1
	H15915	AACTGCAGTCATCTCCGGTTTACAAGAC	1
	14764F	TCGTCGCTGCTCTAGTTGTG	This Study
	15042R	GGATGGCGTAGGCGAATAGA	This Study
	14947F	TCCTAGGAGACCCCGACAAC	This Study
	15124R	TGGAGGTGAGGGATTAGGGC	This Study
	14979F	AACCCCTTAGTCACCCCTCC	This Study
	15222R	GCCCTCCGATTCAGGTTAGG	This Study
	15065F	AGGAGGAGTTTTGGCACTAGC	This Study
	15350R	AGGGCTATTTCTTCATTTGAGTAGT	This Study
	15299F	ACCACTAACAAGCCTATTTGAAAAC	This Study
	15528R	GCAGGTGCGGTTGTTGAGTA	This Study
**D-loop**	15381F	AAACCGAAAACGGAGCACCC	This Study
	15648R	ATGGGGACAGGGTCTTAATGT	This Study
	cytb9FPygmy	AGCCACACTCACACGATTCT	This Study
	cytb9RPygmy	AGGGTCCCCTAAGAGGTCAG	This Study
	cytb10FPygmy	AGTTTTATTCTCCCCTGACCTCT	This Study
	15628F	ACATTAAGACCCTGTCCCCA	This Study
**ND-4**	10169F	GTATCCAACACCTACGGCAT	This Study
	10336F	CACCTACTCCACCAACCTAT	This Study
	10405R	GGGGTAGATAGGGTGTCGGA	This Study
	10514F	TCACCTTACAAACACTCTTAGTT	This Study
	10566R	TAAGATCAATTCGGAGGCTGT	This Study
	10710F	CTACACCCAAAACCTCGC	This Study
	10742R	AGATTTAGTGACCCTGCGAG	This Study
	10926R	TAGGAGGATAGCTGCTAAAACT	This Study
	ND4-2FPygmy	CTATCCCCCTCCTTGTTCTCAC	This Study
	ND4-2RPygmy	TGTCTGGTTTCCTCATCGGG	This Study
	ND4-3FPygmy	CACCCGATGAGGAAACCAGA	This Study

ND4-3RPygmy	TGGGGAGTCATAAGTGTGTGC	This Study
ND4-4FPygmy	GCACACACTTATGACTCCCCA	This Study
ND4-4RPygmy	TTGTCGTAGGCAGATGGAGC	This Study

Table S3b. Primer Pairs and Annealing Temperatures Used in this Study

**Locus**	**Genus**	**Sample Type:**	**Primer Pair**	**Annealing Temp. (C)**
**CO1**	Both	Modern	5288F-5704R	50
	Both	Historical	5288F - 5509R	50
	Both	Historical	5477F - 5704R	50
	Both	Historical	5605F-5841R	50
	Both	Historical	5821F-6052R	50
	Both	Historical	6002F-6242R	50
**cyt-b**	Both	Modern	L14724-H15149	50
	Both	Modern	L15162-H15915	50
	Both	Modern	L15996-16498	50
	Both	Historical	14764F-15042R	56
	Both	Historical	14947F-15124R	56
	Both	Historical	14979F-15222R	56
	Both	Historical	15065F-15350R	50
	Both	Historical	15299F-15528R	55
**D-loop**	*Nycticebus*	Historical	15381F-15648R	50
	Xanthonycticebus	Historical	cytb9F-pygmy-cytb9R-pygmy	50
	Nycticebus	Historical	15628F-H15915	50
	Xanthonycticebus	Historical	cytb10Fpygmy-H15915	50
**ND4**	Both	Modern	10169F-10566R	50
	Both	Modern	10336F-10926R	50
	Both	Historical	10169F-10405R	50
	Nycticebus	Historical	10336F-10566R	50
	Nycticebus	Historical	10514F-10752R	50
	Nycticebus	Historical	10710F-10926R	50
	Xanthonycticebus	Historical	nd4-2Fpygmy-nd4-2Rpygmy	50
	Xanthonycticebus	Historical	nd4-3Fpygmy-nd4-3Rpygmy	50
	Xanthonycticebus	Historical	nd4-4Fpygmy-nd4-4Rpygmy	50

References

1. Irwin, D.M.; Kocher, T.D.; Wilson, A.C. Evolution of the cytochrome b gene of mammals. Journal of Molecular Evolution. 1991, 32, 128-144.
