# Supplementary material for: Molecular Phylogenetic Relationships and Unveiling Novel Genetic Diversity among Slow and Pygmy Lorises, including Resurrection of Xanthonycticebus intermedius
Source: Genes (Basel). 2023 Mar 3;14(3):643. doi: 10.3390/genes14030643 (PMC10048081; doi:10.3390/genes14030643)
Supplement: Supplementary file 1 [file genes-14-00643-s001.zip › Table S4.pdf]

Table S4a. Mean pairwise genetic distances within and between subclades of clade 1

	1-1	1-h	1-m	1-nb	1-tb
1-1	**0.006**	0.0074	0.0059	0.0052	0.0068
1-h		**0.0010**	0.0083	0.0077	0.0111
1-m			**0.0018**	0.0071	0.0064
1-nb				**0.0040**	0.0067
1-tb					**0.0038**

Table S4b. Pairwise genetic distances of all individual sequences included in the phylogenetic analysis

		1	2	3	4	5	6	7	8	9	10	11	12	13	14	15	16	17	18	19	20	21	22	23	24	25	26	27	28	29	30
1	5 AMNH102027	-																													
2	26 AMNH20870	0.0543	-																												
3	27 AMNH240010	0.0529	0.0056	-																											
4	29 AMNH112991	0.0507	0.0050	0.0077	-																										
5	30 AMNH112990	0.0514	0.0043	0.0066	0.0028	-																									
6	34 AMNH16616	0.0655	0.0434	0.0445	0.0421	0.0420	-																								
7	40 AMNH102845	0.0021	0.0468	0.0478	0.0478	0.0469	0.0616	-																							
8	41 AMNH101509	0.0011	0.0460	0.0460	0.0429	0.0429	0.0628	0.0033	-																						
9	59 AMNH106013	0.0642	0.0455	0.0453	0.0436	0.0436	0.0267	0.0603	0.0566	-																					
10	60 AMNH106012	0.0595	0.0381	0.0480	0.0425	0.0432	0.0255	0.0602	0.0596	0.0290	-																				
11	61 AMNH17113	0.0672	0.0496	0.0502	0.0477	0.0487	0.0066	0.0607	0.0587	0.0321	0.0267	-																			
12	63 AMNH32649	0.0681	0.0472	0.0479	0.0456	0.0463	0.0078	0.0602	0.0598	0.0305	0.0253	0.0014	-																		
13	66 NMNH300000	0.0536	0.0013	0.0053	0.0043	0.0048	0.0447	0.0498	0.0494	0.0450	0.0367	0.0500	0.0483	-																	
14	70 NMNH317188	0.0727	0.0513	0.0567	0.0553	0.0562	0.0314	0.0659	0.0612	0.0081	0.0332	0.0387	0.0358	0.0535	-																
15	77 NMNH198857	0.0687	0.0448	0.0459	0.0439	0.0446	0.0221	0.0613	0.0625	0.0288	0.0054	0.0289	0.0285	0.0441	0.0333	-															
16	79 NMNH142239	0.0717	0.0507	0.0519	0.0498	0.0505	0.0063	0.0666	0.0673	0.0322	0.0236	0.0091	0.0085	0.0500	0.0376	0.0274	-														
17	82 NMNH142237	0.0679	0.0493	0.0467	0.0473	0.0484	0.0077	0.0613	0.0598	0.0311	0.0255	0.0023	0.0000	0.0522	0.0344	0.0280	0.0083	-													
18	83 NMNH142232	0.0690	0.0524	0.0471	0.0480	0.0492	0.0070	0.0611	0.0608	0.0315	0.0253	0.0114	0.0037	0.0531	0.0378	0.0281	0.0078	0.0102	-												

19	87 NMNH292553	0.0706	0.0489	0.0524	0.0497	0.0507	0.0277	0.0630	0.0576	0.0046	0.0289	0.0330	0.0336	0.0487	0.0071	0.0304	0.0347	0.0336	0.0337	-											
20	88 NMNH521836	0.0024	0.0665	0.0554	0.0658	0.0658	0.0602	0.0044	0.0022	0.0743	0.0596	0.0750	0.0668	0.0714	0.0809	0.0710	0.0739	0.0790	0.0814	0.0762	-										
21	91 NMNH258870	0.0545	0.0088	0.0127	0.0084	0.0084	0.0456	0.0496	0.0451	0.0460	0.0466	0.0517	0.0492	0.0092	0.0590	0.0470	0.0536	0.0490	0.0499	0.0537	0.0682	-									
22	93 NMNH296513	0.0520	0.0064	0.0028	0.0044	0.0050	0.0457	0.0442	0.0426	0.0471	0.0406	0.0505	0.0494	0.0054	0.0541	0.0471	0.0531	0.0503	0.0528	0.0505	0.0643	0.0082	-								
23	95 NMNH14290	0.0686	0.0174	0.0159	0.0225	0.0243	0.00000*	0.00000*	0.00000*	0.0436	0.00000*	0.0561	0.0553	0.0272	0.0470	0.0712	0.0616	0.0453	0.0657	0.0554	0.1061	0.0244	0.0145	-							
24	99 NMNH300015	0.0499	0.0062	0.0077	0.0052	0.0042	0.0422	0.0452	0.0417	0.0437	0.0446	0.0483	0.0463	0.0055	0.0549	0.0462	0.0520	0.0479	0.0487	0.0502	0.0654	0.0098	0.0056	0.0230	-						
25	101 NMNH535153	0.0529	0.0056	0.0022	0.0084	0.0075	0.0442	0.0478	0.0461	0.0477	0.0480	0.0517	0.0477	0.0060	0.0590	0.0461	0.0519	0.0500	0.0511	0.0538	0.0682	0.0122	0.0025	0.0225	0.0080	-					
26	102 NMNH355064	0.0522	0.0068	0.0044	0.0094	0.0094	0.0455	0.0485	0.0450	0.0466	0.0481	0.0532	0.0484	0.0067	0.0581	0.0469	0.0528	0.0505	0.0522	0.0533	0.0691	0.0131	0.0025	0.0231	0.0098	0.0056	-				
27	105 NMNH84389	0.0559	0.0028	0.0040	0.0042	0.0049	0.0433	0.0514	0.0511	0.0461	0.0354	0.0513	0.0489	0.0014	0.0526	0.0421	0.0499	0.0511	0.0539	0.0493	0.0710	0.0084	0.0049	0.0180	0.0056	0.0042	0.0056	-			
28	108 NMNH114151	0.0519	0.0063	0.0060	0.0030	0.0042	0.0423	0.0456	0.0427	0.0432	0.0362	0.0456	0.0472	0.0054	0.0508	0.0447	0.0507	0.0455	0.0477	0.0468	0.0634	0.0018	0.0057	0.0134	0.0047	0.0059	0.0071	0.0056	-		
29	109 NMNH355347	0.0543	0.0025	0.0079	0.0053	0.0065	0.0411	0.0474	0.0450	0.0438	0.0362	0.0462	0.0472	0.0013	0.0507	0.0440	0.0500	0.0460	0.0483	0.0467	0.0645	0.0082	0.0063	0.0163	0.0070	0.0071	0.0082	0.0021	0.0059	-	
30	112 NMNH488075	0.0537	0.0013	0.0091	0.0086	0.0086	0.0423	0.0512	0.0452	0.0485	0.0433	0.0503	0.0465	0.0012	0.0555	0.0440	0.0499	0.0511	0.0530	0.0515	0.0682	0.0113	0.0051	0.0239	0.0092	0.0081	0.0097	0.0014	0.0095	0.0059	-
31	113 NMNH270595	0.0552	0.0088	0.0106	0.0071	0.0076	0.0443	0.0503	0.0471	0.0458	0.0439	0.0513	0.0485	0.0074	0.0568	0.0432	0.0505	0.0484	0.0506	0.0518	0.0665	0.0116	0.0094	0.0204	0.0091	0.0116	0.0132	0.0077	0.0071	0.0083	0.0118
32	114 NMNH267400	0.0552	0.0081	0.0110	0.0066	0.0070	0.0443	0.0504	0.0472	0.0447	0.0439	0.0502	0.0484	0.0072	0.0562	0.0431	0.0505	0.0484	0.0493	0.0507	0.0691	0.0108	0.0081	0.0263	0.0084	0.0108	0.0117	0.0062	0.0059	0.0070	0.0102
33	116 NMNH283915	0.0530	0.0019	0.0059	0.0059	0.0059	0.0423	0.0459	0.0452	0.0440	0.0381	0.0462	0.0458	0.0018	0.0517	0.0432	0.0491	0.0474	0.0488	0.0473	0.0644	0.0087	0.0057	0.0254	0.0065	0.0054	0.0070	0.0021	0.0065	0.0030	0.0038
34	119 NMNH488078	0.0537	0.0013	0.0059	0.0048	0.0059	0.0423	0.0467	0.0452	0.0440	0.0372	0.0462	0.0465	0.0012	0.0516	0.0440	0.0499	0.0474	0.0488	0.0473	0.0654	0.0076	0.0051	0.0239	0.0065	0.0054	0.0059	0.0014	0.0054	0.0018	0.0038
35	120 NMNH488076	0.0537	0.0019	0.0065	0.0059	0.0060	0.0423	0.0466	0.0452	0.0453	0.0380	0.0474	0.0465	0.0018	0.0528	0.0440	0.0499	0.0484	0.0499	0.0485	0.0660	0.0087	0.0057	0.0254	0.0065	0.0065	0.0081	0.0021	0.0065	0.0029	0.0049
36	122 NMNH488079	0.0537	0.0013	0.0052	0.0054	0.0054	0.0423	0.0459	0.0452	0.0447	0.0381	0.0468	0.0465	0.0012	0.0522	0.0440	0.0499	0.0479	0.0494	0.0479	0.0649	0.0081	0.0051	0.0239	0.0059	0.0049	0.0064	0.0014	0.0060	0.0024	0.0032
37	124 FMNH99616	0.0530	0.0069	0.0077	0.0061	0.0052	0.0410	0.0462	0.0440	0.0442	0.0440	0.0487	0.0464	0.0067	0.0548	0.0447	0.0505	0.0479	0.0498	0.0507	0.0667	0.0098	0.0063	0.0245	0.0056	0.0080	0.0098	0.0063	0.0053	0.0077	0.0081
38	128 FMNH85926	0.0697	0.0482	0.0534	0.0515	0.0525	0.0265	0.0623	0.0578	0.0040	0.0296	0.0342	0.0330	0.0499	0.0066	0.0302	0.0346	0.0317	0.0342	0.0025	0.0771	0.0553	0.0510	0.0426	0.0520	0.0553	0.0543	0.0491	0.0472	0.0472	0.0517
39	131 FMNH98478	0.0551	0.0019	0.0057	0.0044	0.0050	0.0413	0.0468	0.0460	0.0440	0.0362	0.0483	0.0465	0.0007	0.0500	0.0433	0.0492	0.0480	0.0511	0.0470	0.0666	0.0082	0.0057	0.0164	0.0056	0.0050	0.0063	0.0021	0.0057	0.0019	0.0006
40	133 FMNH108856	0.0688	0.0487	0.0488	0.0490	0.0501	0.0278	0.0612	0.0566	0.0019	0.0308	0.0339	0.0337	0.0504	0.0059	0.0309	0.0354	0.0328	0.0348	0.0024	0.0790	0.0512	0.0516	0.0437	0.0485	0.0523	0.0506	0.0497	0.0478	0.0477	0.0527
41	134 FMNH129502	0.0706	0.0489	0.0477	0.0481	0.0486	0.0279	0.0589	0.0577	0.0007	0.0291	0.0336	0.0336	0.0487	0.0050	0.0304	0.0348	0.0347	0.0348	0.0025	0.0805	0.0506	0.0507	0.0553	0.0480	0.0499	0.0499	0.0493	0.0481	0.0481	0.0480
42	aDNA716	0.0510	0.0043	0.0071	0.0009	0.0019	0.0419	0.0471	0.0431	0.0436	0.0434	0.0476	0.0455	0.0036	0.0552	0.0438	0.0497	0.0472	0.0479	0.0496	0.0659	0.0084	0.0037	0.0213	0.0042	0.0074	0.0093	0.0034	0.0029	0.0052	0.0075
43	AJ309867	0.0524	0.0049	0.0038	0.0074	0.0065	0.0429	0.0470	0.0451	0.0454	0.0459	0.0500	0.0469	0.0048	0.0571	0.0452	0.0511	0.0478	0.0491	0.0521	0.0673	0.0112	0.0018	0.0213	0.0070	0.0037	0.0065	0.0034	0.0041	0.0052	0.0069
44	KC757405	0.0524	0.0049	0.0049	0.0093	0.0084	0.0428	0.0462	0.0451	0.0466	0.0486	0.0501	0.0469	0.0048	0.0571	0.0452	0.0511	0.0483	0.0497	0.0521	0.0663	0.0103	0.0018	0.0213	0.0089	0.0047	0.0065	0.0034	0.0047	0.0058	0.0064
45	KC977312	0.0518	0.0049	0.0099	0.0065	0.0056	0.0419	0.0490	0.0431	0.0460	0.0476	0.0501	0.0463	0.0048	0.0571	0.0446	0.0505	0.0494	0.0498	0.0522	0.0688	0.0113	0.0044	0.0240	0.0061	0.0103	0.0121	0.0041	0.0048	0.0071	0.0097
46	KX397281	0.1137	0.1120	0.0972	0.1072	0.1072	0.0870	0.0918	0.0962	0.1034	0.0890	0.1105	0.1129	0.1209	0.1092	0.1132	0.1197	0.1189	0.1196	0.1046	0.1155	0.1091	0.1127	0.1614	0.1073	0.1086	0.1086	0.1168	0.1099	0.1109	0.1162

47	L0	0.1124	0.1132	0.1025	0.1162	0.1168	0.0871	0.0954	0.0983	0.1128	0.0951	0.1192	0.1128	0.1186	0.1205	0.1103	0.1170	0.1227	0.1241	0.1157	0.1298	0.1187	0.1135	0.1589	0.1174	0.1162	0.1156	0.1141	0.1136	0.1147	0.1179
48	L2	0.1161	0.1154	0.1063	0.1181	0.1187	0.0883	0.0997	0.0997	0.1159	0.0972	0.1213	0.1164	0.1205	0.1225	0.1125	0.1191	0.1246	0.1262	0.1179	0.1317	0.1194	0.1156	0.1599	0.1194	0.1181	0.1175	0.1170	0.1158	0.1169	0.1198
49	L6	0.1160	0.1189	0.1046	0.1213	0.1219	0.0882	0.0997	0.0997	0.1206	0.0951	0.1255	0.1146	0.1237	0.1262	0.1108	0.1189	0.1283	0.1306	0.1222	0.1380	0.1225	0.1193	0.1697	0.1225	0.1213	0.1207	0.1207	0.1194	0.1205	0.1229
50	L21	0.0517	0.0053	0.0031	0.0012	0.0018	0.0442	0.0480	0.0473	0.0442	0.0367	0.0490	0.0481	0.0042	0.0529	0.0447	0.0506	0.0508	0.0521	0.0486	0.0698	0.0062	0.0047	0.0226	0.0025	0.0043	0.0049	0.0042	0.0028	0.0054	0.0043
51	L34	0.0524	0.0066	0.0038	0.0024	0.0030	0.0454	0.0490	0.0484	0.0457	0.0377	0.0504	0.0488	0.0055	0.0541	0.0454	0.0513	0.0521	0.0535	0.0499	0.0710	0.0074	0.0061	0.0239	0.0037	0.0055	0.0061	0.0056	0.0041	0.0067	0.0055
52	L40	0.1574	0.1559	0.1456	0.1575	0.1592	0.00000*	0.00000*	0.00000*	0.1551	0.00000*	0.1586	0.1571	0.1610	0.1538	0.1572	0.1653	0.1614	0.1721	0.1538	0.1801	0.1612	0.1560	0.1539	0.1607	0.1565	0.1562	0.1611	0.1618	0.1591	0.1589
53	L41	0.1203	0.1268	0.1178	0.1284	0.1290	0.1116	0.1058	0.1101	0.1263	0.1163	0.1327	0.1273	0.1302	0.1327	0.1257	0.1324	0.1350	0.1382	0.1287	0.1362	0.1297	0.1266	0.1630	0.1284	0.1284	0.1272	0.1283	0.1274	0.1285	0.1301
54	L42	0.1171	0.1252	0.1160	0.1264	0.1270	0.1086	0.1021	0.1060	0.1238	0.1126	0.1298	0.1241	0.1282	0.1289	0.1225	0.1291	0.1311	0.1351	0.1258	0.1329	0.1276	0.1250	0.1579	0.1264	0.1264	0.1252	0.1267	0.1258	0.1269	0.1281
55	LANGKAWI	0.0600	0.0071	0.0039	0.0027	0.0037	0.0411	0.0630	0.0637	0.0466	0.0306	0.0510	0.0499	0.0071	0.0572	0.0439	0.0540	0.0526	0.0584	0.0522	0.0850	0.0073	0.0053	0.0211	0.0037	0.0046	0.0055	0.0062	0.0043	0.0062	0.0053
56	N23	0.0000	*0.00000	*0.00000	*0.00000	*0.00000	*0.00000*	0.00000*	0.00000*	0.0000	*0.00000*	0.0000	*0.00000	*0.00000	*	0.0000	*0.00000	*0.00000	*0.00000	*0.00000	*0.00000*	0.00000*	0.00000*	0.0000	*0.00000*	0.0000	*0.00000	*0.00000	*0.00000	0.0000	*0.00000
57	N24	0.1170	0.1251	0.1165	0.1269	0.1274	0.1092	0.1017	0.1057	0.1236	0.1128	0.1297	0.1248	0.1295	0.1292	0.1231	0.1298	0.1316	0.1351	0.1255	0.1329	0.1287	0.1249	0.1586	0.1268	0.1268	0.1256	0.1267	0.1254	0.1267	0.1286
58	NC40292	0.0540	0.0013	0.0110	0.0098	0.0093	0.0421	0.0473	0.0454	0.0448	0.0434	0.0487	0.0464	0.0018	0.0548	0.0440	0.0498	0.0484	0.0493	0.0492	0.0668	0.0131	0.0051	0.0258	0.0098	0.0102	0.0121	0.0014	0.0060	0.0024	0.0038
59	NCOU5751	0.0000	*0.00000	*0.00000	*0.00000	*0.00000	*0.00000*	0.00000*	0.00000*	0.0000	*0.00000*	0.0000	*0.00000	*0.00000	*	0.0000	*0.00000	*0.00000	*0.00000	*0.00000	*0.00000*	0.00000*	0.00000*	0.0000	*0.00000*	0.0000	*0.00000	*0.00000	*	0.0000	*0.00000
60	N3NJav	0.0000	*0.00000	*0.00000	*0.00000	*0.00000	*0.00000*	0.00000*	0.00000*	0.0000	*0.00000*	0.0000	*0.00000	*0.00000	*	0.0000	*0.00000	*0.00000	*0.00000	*0.00000	*0.00000*	0.00000*	0.00000*	0.0000	*0.00000*	0.0000	*0.00000	*0.00000	*	0.0000	*0.00000
61	NJAV	0.0000	*0.00000	*0.00000	*0.00000	*0.00000	*0.00000*	0.00000*	0.00000*	0.0000	*0.00000*	0.0000	*0.00000	*0.00000	*	0.0000	*0.00000	*0.00000	*0.00000	*0.00000	*0.00000*	0.00000*	0.00000*	0.0000	*0.00000*	0.0000	*0.00000	*0.00000	*	0.0000	*0.00000
62	ZSM3	0.0000	*0.00000	*0.00000	*0.00000	*0.00000	*0.00000*	0.00000*	0.00000*	0.0000	*0.00000*	0.0000	*0.00000	*0.00000	*	0.0000	*0.00000	*0.00000	*0.00000	*0.00000	*0.00000*	0.00000*	0.00000*	0.0000	*0.00000*	0.0000	*0.00000	*0.00000	*	0.0000	*0.00000
63	KC757396 Gmoholi	0.2307	0.2347	0.2256	0.2306	0.2323	0.2254	0.2174	0.2279	0.2369	0.2157	0.2298	0.2262	0.2328	0.2283	0.2266	0.2265	0.2283	0.2333	0.2303	0.2316	0.2293	0.2327	0.2463	0.2305	0.2299	0.2305	0.2338	0.2319	0.2338	0.2318
64	NC012763 Ltardigradus	0.1989	0.2029	0.2004	0.2041	0.2047	0.2153	0.2007	0.1949	0.2023	0.2073	0.2023	0.2052	0.2065	0.2035	0.2068	0.2096	0.2053	0.2079	0.2048	0.2018	0.2041	0.2039	0.2022	0.2041	0.2046	0.2052	0.2060	0.2052	0.2064	0.2054

	31	32	33	34	35	36	37	38	39	40	41	42	43	44	45	46	47	48	49	50	51	52	53	54	55	56	57	58	59	60	61	62	63	64
31	-																																	
32	0.0010	-																																
33	0.0089	0.0076	-																															
34	0.0077	0.0065	0.0011	-																														

35	0.0089	0.0076	0.0022	0.0022	-																					
36	0.0083	0.0070	0.0005	0.0005	0.0016	-																				
37	0.0096	0.0084	0.0054	0.0054	0.0065	0.0048	-																			
38	0.0532	0.0525	0.0479	0.0479	0.0490	0.0485	0.0520	-																		
39	0.0082	0.0069	0.0013	0.0006	0.0013	0.0006	0.0050	0.0468	-																	
40	0.0502	0.0501	0.0490	0.0490	0.0501	0.0495	0.0501	0.0011	0.0474	-																
41	0.0507	0.0493	0.0474	0.0480	0.0487	0.0480	0.0487	0.0019	0.0470	0.0025	-															
42	0.0071	0.0066	0.0048	0.0048	0.0048	0.0043	0.0051	0.0515	0.0037	0.0490	0.0472	-														
43	0.0100	0.0088	0.0042	0.0042	0.0043	0.0037	0.0079	0.0533	0.0043	0.0500	0.0491	0.0063	-													
44	0.0131	0.0117	0.0037	0.0037	0.0048	0.0032	0.0079	0.0533	0.0043	0.0506	0.0491	0.0075	0.0048	-												
45	0.0101	0.0098	0.0070	0.0070	0.0070	0.0064	0.0061	0.0543	0.0043	0.0512	0.0479	0.0051	0.0090	0.0096	-											
46	0.1065	0.1100	0.1134	0.1145	0.1146	0.1140	0.1076	0.1083	0.1127	0.1147	0.1157	0.1095	0.1092	0.1089	0.1095	-										
47	0.1156	0.1193	0.1173	0.1179	0.1185	0.1179	0.1180	0.1187	0.1141	0.1186	0.1156	0.1169	0.1151	0.1155	0.1172	0.0029	-									
48	0.1178	0.1212	0.1192	0.1198	0.1204	0.1198	0.1200	0.1206	0.1162	0.1205	0.1178	0.1202	0.1187	0.1188	0.1205	0.0162	0.0164	-								
49	0.1228	0.1244	0.1224	0.1230	0.1236	0.1229	0.1225	0.1244	0.1199	0.1243	0.1221	0.1215	0.1200	0.1200	0.1219	0.0186	0.0188	0.0057	-							
50	0.0061	0.0055	0.0049	0.0043	0.0049	0.0043	0.0037	0.0492	0.0046	0.0498	0.0485	0.0018	0.0049	0.0049	0.0007	0.1157	0.1167	0.1200	0.1213	-						
51	0.0074	0.0067	0.0061	0.0055	0.0061	0.0055	0.0049	0.0504	0.0060	0.0510	0.0499	0.0025	0.0056	0.0056	0.0014	0.1164	0.1175	0.1208	0.1222	0.0007	-					
52	0.1616	0.1626	0.1576	0.1590	0.1604	0.1589	0.1639	0.1523	0.1593	0.1505	0.1539	0.1585	0.1574	0.1574	0.1612	0.0230	0.0202	0.0034	0.0167	0.1600	0.1617	-				
53	0.1294	0.1316	0.1296	0.1302	0.1308	0.1302	0.1303	0.1309	0.1278	0.1295	0.1287	0.1229	0.1211	0.1215	0.1232	0.0082	0.0091	0.0227	0.0255	0.1227	0.1235	0.0212	-			
54	0.1277	0.1295	0.1275	0.1281	0.1288	0.1281	0.1282	0.1270	0.1262	0.1257	0.1257	0.1207	0.1189	0.1193	0.1211	0.0085	0.0087	0.0211	0.0235	0.1206	0.1213	0.0176	0.0032	-		
55	0.0075*	0.0055*	0.0062*	0.0053*	0.0062	0.0053	0.0045	0.0518*	0.0071	0.0535*	0.0532*	0.0019*	0.0027	0.0028	0.0037	0.1274	0.1257	0.1285	0.1350	0.0028	0.0046	0.1592	0.1430	0.1410	-	
56	0.00000	0.00000	0.00000	0.00000*	0.00000*	0.00000*	0.0000	0.00000*	0.0000	0.00000	0.00000	0.11974	0.1163	0.1172	0.1189	0.0114	0.0148	0.0017	0.0010	0.1181	0.1182	0.00000*	0.0121	0.0135	0.0000	* -

57	0.1275	0.1299	0.1280	0.1286	0.1292	0.1286	0.1287	0.1275	0.1261	0.1262	0.1256	0.1275	0.1268	0.1269	0.1287	0.0134	0.0110	0.0263	0.0325	0.1282	0.1295	0.0190	0.0036	0.0006	0.1416	0.0000	-							
58	0.0106*	0.0098*	0.0011*	0.0011*	0.0022	0.0005	0.0088	0.0510*	0.0006	0.0501*	0.0480*	0.0117*	0.0117	0.0117	0.0135	0.1113	0.1194	0.1209	0.1223	0.0099	0.0106	0.1603	0.1254	0.1232	0.0063	0.1197*	0.1294	-*						
59	0.00000*	0.00000*	0.00000*	0.00000**	0.00000*	0.00000*	0.0000	0.00000**	0.0000	0.00000*	0.00000*	0.07489*	0.0705	0.0741	0.0757	0.1220	0.1257	0.1216	0.1245	0.0748	0.0748	0.00000*	0.1227	0.1211	0.0000	0.12129*	0.0000	0.07690*	-					
60	0.00000*	0.00000*	0.00000*	0.00000**	0.00000*	0.00000*	0.0000	0.00000**	0.0000	0.00000*	0.00000*	0.02623*	0.0272	0.0272	0.0271	0.1158	0.1178	0.1173	0.1175	0.0262	0.0262	0.00000*	0.1176	0.1151	0.0000	0.11507*	0.0000	0.03005*	0.0754	-				
61	0.00000*	0.00000*	0.00000*	0.00000**	0.00000*	0.00000*	0.0000	0.00000**	0.0000	0.00000*	0.00000*	0.02530*	0.0263	0.0263	0.0261	0.1167	0.1186	0.1182	0.1175	0.0253	0.0253	0.00000*	0.1184	0.1160	0.0000	0.11595*	0.0000	0.02913*	0.0763	0.0009	-			
62	0.00000	0.00000	0.00000	0.00000*	0.00000*	0.00000*	0.0000	0.00000*	0.0000	0.00000	0.00000	0.07582	0.0714	0.0750	0.0767	0.1230	0.1267	0.1217	0.1247	0.0758	0.0758	0.00000*	0.1237	0.1221	0.0000	0.12215	0.0000	0.07952	0.0044	0.0781	0.0790	-		
63	0.2357	0.2348	0.2324	0.2318	0.2312	0.2318	0.2312	0.2295	0.2327	0.2283	0.2301	0.2145	0.2152	0.2155	0.2148	0.2144	0.2147	0.2151	0.2185	0.2143	0.2144	0.2557	0.2106	0.2104	0.2471	0.1956	0.2172	0.2173	0.1863	0.1783	0.1792	0.1864	-	
64	0.2069	0.2066	0.2061	0.2055	0.2055	0.2054	0.2040	0.2023	0.2045	0.2012	0.2039	0.1918	0.1918	0.1933	0.1925	0.2042	0.2035	0.2017	0.2030	0.1929	0.1930	0.2043	0.2048	0.2025	0.2098	0.1826	0.2151	0.1957	0.1851	0.1760	0.1769	0.1860	0.2015	-
