# Supplementary figures and images for: Molecular Phylogenetic Relationships and Unveiling Novel Genetic Diversity among Slow and Pygmy Lorises, including Resurrection of Xanthonycticebus intermedius
Source: Genes (Basel). 2023 Mar 3;14(3):643. doi: 10.3390/genes14030643 (PMC10048081; doi:10.3390/genes14030643)

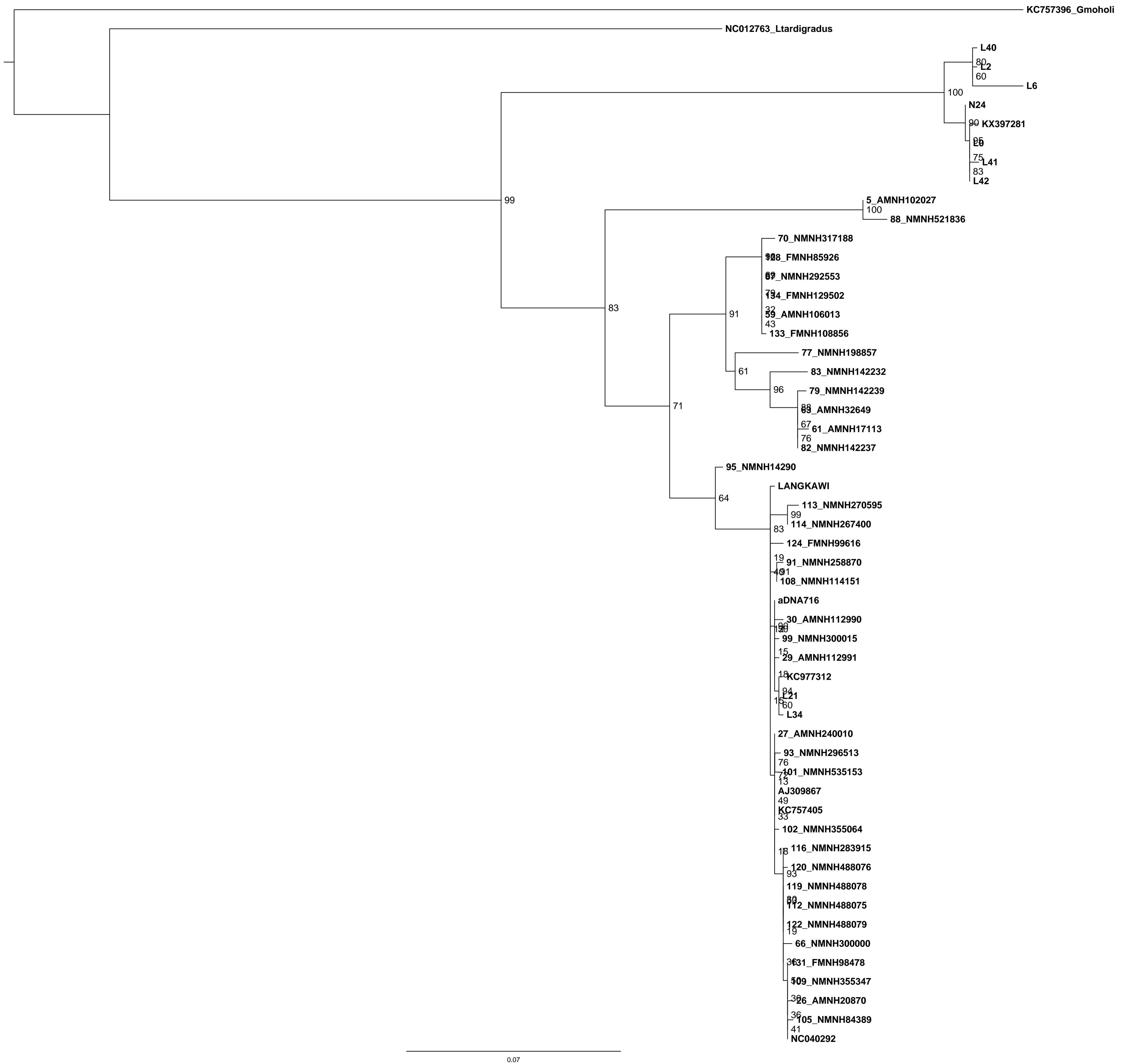

Supplement: Supplementary file 1 [file genes-14-00643-s001.zip › Figure S1. ND4Tree.pdf]

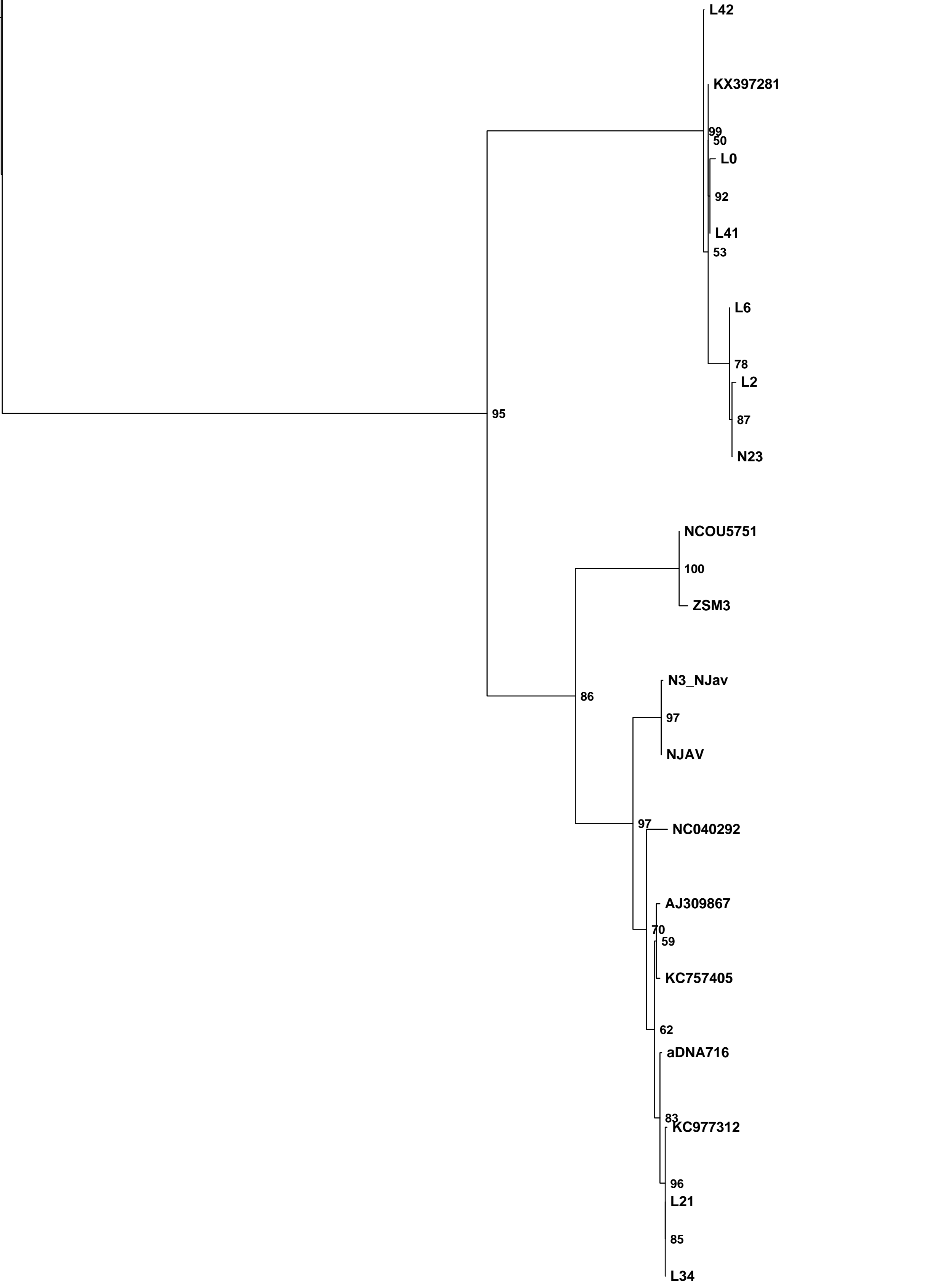

Supplement: Supplementary file 1 [file genes-14-00643-s001.zip › Figure S2. CytbTree.pdf]

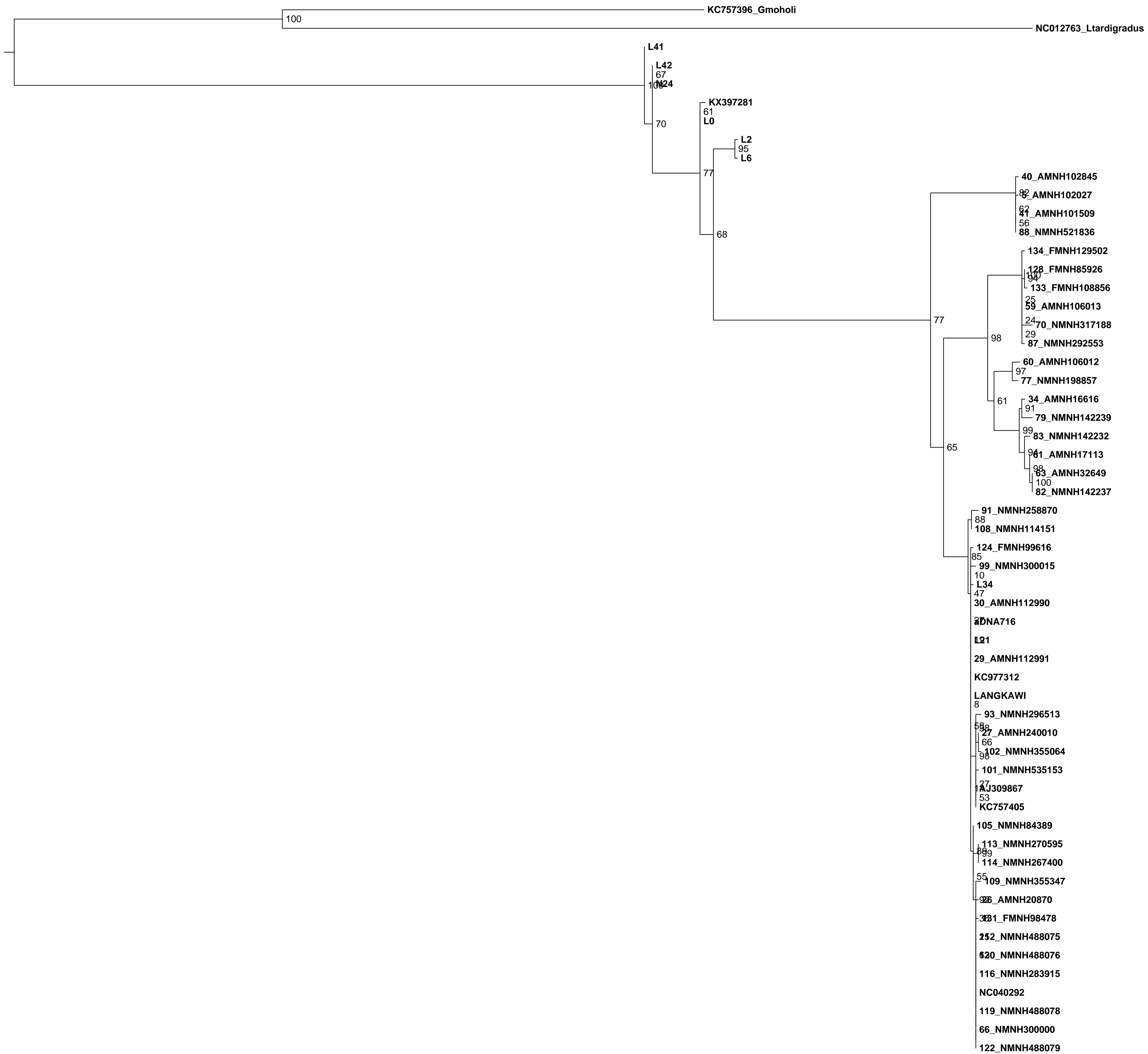

Supplement: Supplementary file 1 [file genes-14-00643-s001.zip › Figure S3. COITree.pdf]

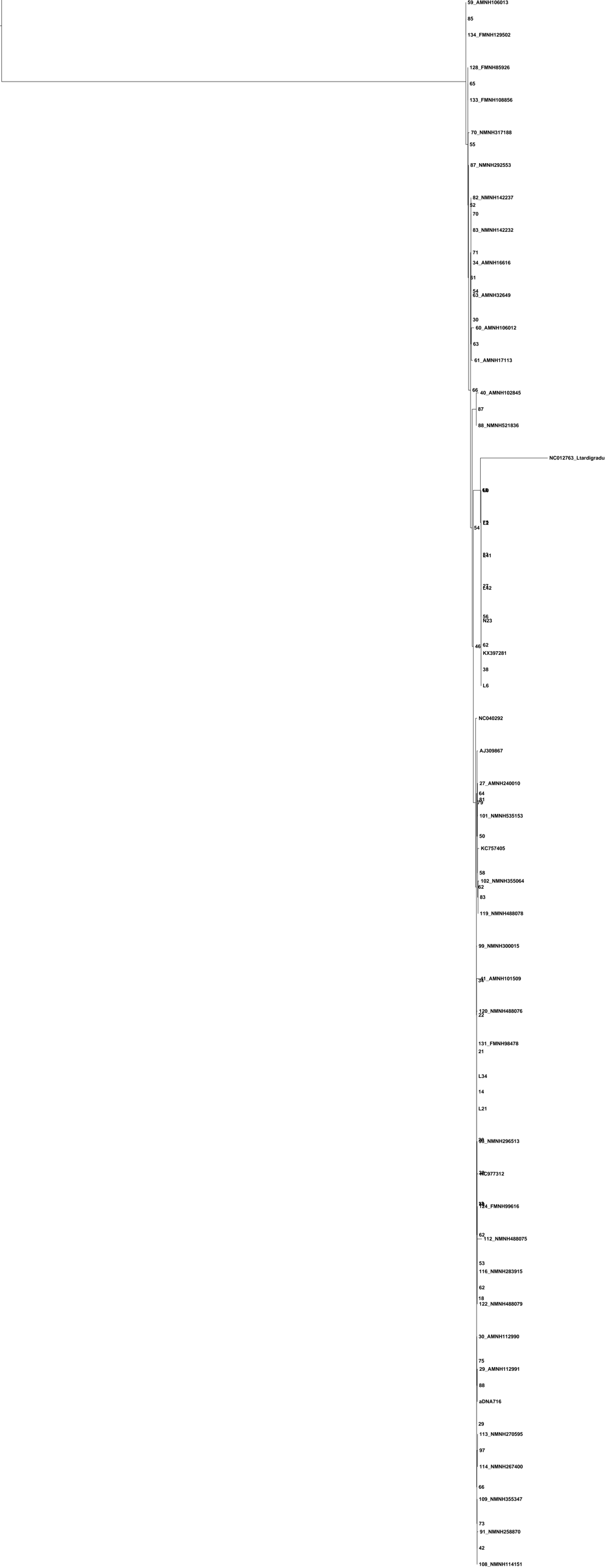

Supplement: Supplementary file 1 [file genes-14-00643-s001.zip › Figure S4. DloopTree.pdf]

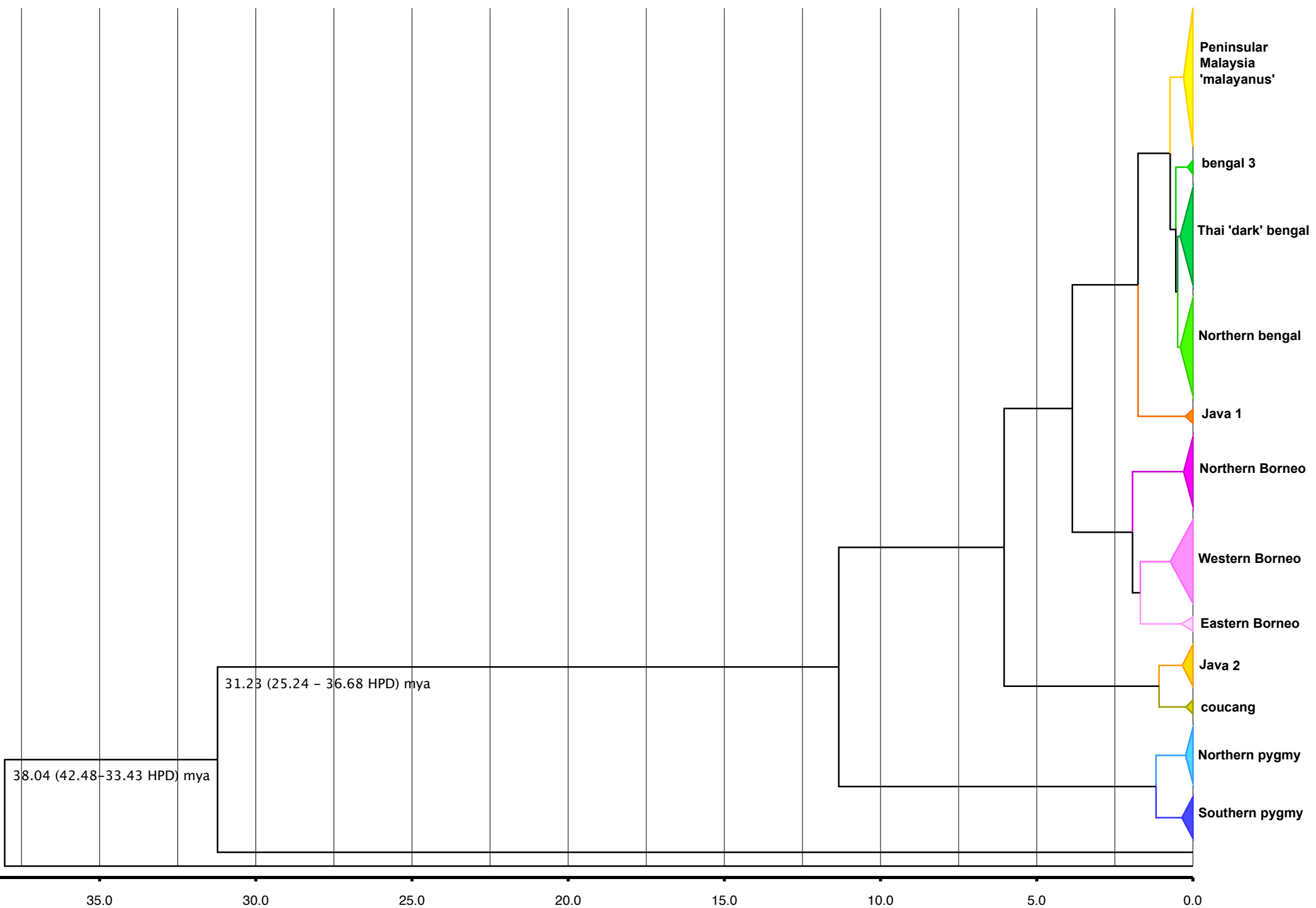

Supplement: Supplementary file 1 [file genes-14-00643-s001.zip › Figure S5. BEAST.pdf]

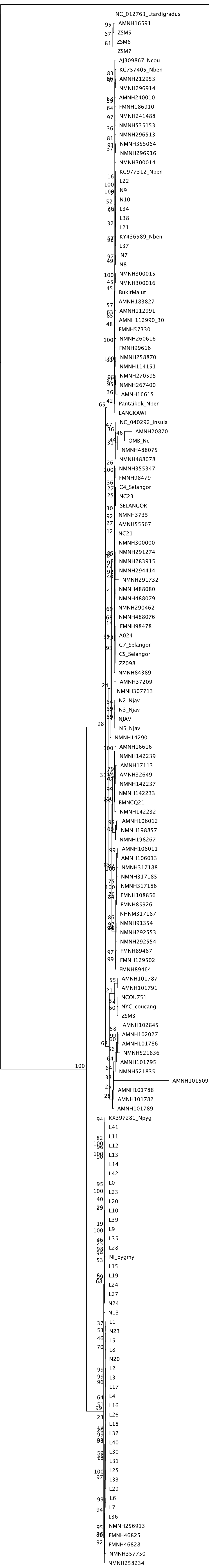

Supplement: Supplementary file 1 [file genes-14-00643-s001.zip › Figure S6. Allsamples_ML_tree.pdf]

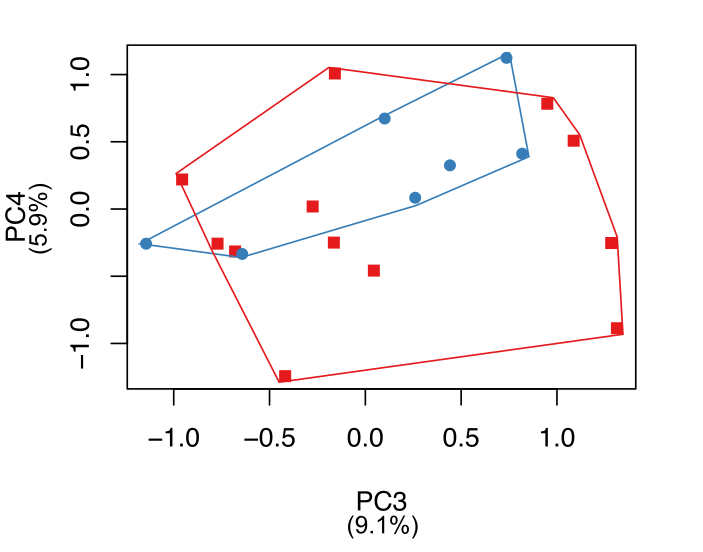

Supplement: Supplementary file 1 [file genes-14-00643-s001.zip › Figure S7. PCA34.png]
